# Will Your Next Therapist Be a Robot?—A Review of the Advancements in Robotic Upper Extremity Rehabilitation

**DOI:** 10.3390/s23115054

**Published:** 2023-05-25

**Authors:** Raouf Fareh, Ammar Elsabe, Mohammed Baziyad, Tunajjina Kawser, Brahim Brahmi, Mohammad H. Rahman

**Affiliations:** 1 Department of Electrical Engineering, University of Sharjah, Sharjah 27272, United Arab Emirates; 2 Department of Computer Engineering, University of Sharjah, Sharjah 27272, United Arab Emirates; 3 Research Institute of Sciences and Engineering (RISE), University of Sharjah, Sharjah 27272, United Arab Emirates; 4 Anatomy Department, Shaheed Tajuddin Ahmad Medical College, Gazipur 1700, Bangladesh; 5 Department of Electrical Engineering, College of Ahuntsic, Montreal, QC H2M 1Y8, Canada; 6 Mechanical Engineering, University of Wisconsin Milwaukee, Milwaukee, WI 53212, USA

**Keywords:** exoskeleton, rehabilitation, review, robots, upper extremity

## Abstract

Several recent studies have indicated that upper extremity injuries are classified as a top common workplace injury. Therefore, upper extremity rehabilitation has become a leading research area in the last few decades. However, this high number of upper extremity injuries is viewed as a challenging problem due to the insufficient number of physiotherapists. With the recent advancements in technology, robots have been widely involved in upper extremity rehabilitation exercises. Although robotic technology and its involvement in the rehabilitation field are rapidly evolving, the literature lacks a recent review that addresses the updates in the robotic upper extremity rehabilitation field. Thus, this paper presents a comprehensive review of state-of-the-art robotic upper extremity rehabilitation solutions, with a detailed classification of various rehabilitative robots. The paper also reports some experimental robotic trials and their outcomes in clinics.

## 1. Introduction

A study conducted by the Bureau of Labor Statistics agency has revealed that there were more than 4.4 million upper extremity injuries from 1992 to 2018 in the USA, which makes upper extremity injuries the second most common workplace injury in the USA [1]. The main cause of upper extremity impairments is stroke. It has been reported that 70% of stroke survivors have upper extremity disability [2]. Upper extremity injuries can include any injury in the upper part of the body, such as the hand, elbow, arm, and shoulder. It is known that upper limb disabilities following a stroke result from brain injuries [3]. A stroke occurs when blood flow to the brain is disrupted, leading to brain cell damage and sometimes even death. The specific location of the brain injury determines the type and severity of stroke-related disabilities, which can affect speech, mobility, and cognitive function. In the case of upper limb disabilities, stroke can result in weakness, spasticity, or lack of coordination, making it difficult to perform everyday tasks, such as dressing, eating, or using tools. Therefore, effective stroke rehabilitation programs should target the underlying brain injury and aim to promote neural plasticity and functional recovery. Upper extremity rehabilitation has become a top research area due to the increasing number of patients and due to the seriousness of this disorder [4,5].

The primary objective of rehabilitation is to help people restore their physical abilities to improve their performance and help patients return to a “normal” state where they can perform Activities of Daily Living (ADL) [6,7]. Classically, rehabilitation is performed by physical therapists to restore and maintain health using repetitive physical exercises and through patient education.

However, due to the nature of traditional therapy and the ever-increasing shortage of physiotherapists, physical rehabilitation has not been accessible to a large number of people. A recent study conducted by the Chartered Society of Physiotherapy (CSP) reported that the number of newly graduated UK physiotherapists should annually grow by 500 graduates for several years to keep pace with the high demand. Moreover, the UK Charity Muscular Dystrophy has revealed that muscular-impairment patients lack an “on life-improving treatment” experience due to the insufficient number of specialist physiotherapists. Based on the charity’s report, 60% of patients having muscle-wasting problems did not receive appropriate physiotherapy treatment. Furthermore, 40% of UK neuromuscular clinks have declared that they urgently need higher physiotherapists capacity [8] (Note: The UK and the USA’s statistics were used due to competent data collection. However, upper extremity impairments and the shortage of physiotherapists are a worldwide problem).

With the great advancements in technology within the last few decades, researchers have found technology a potential solution to the issues of classical rehabilitation. Indeed, robotics are viewed as the best candidate to adopt in order to overcome the rehabilitation growing challenges. Robots are well known for their abilities to efficiently perform dull, dirty, and dangerous tasks (the triple D’s). Rehabilitation exercises are repetitive and a single session can last for multiple hours. Therefore, researchers have been seriously interested in robotic rehabilitation. Moreover, robots as a versatile tool have been proven to be effective and favored by patients over traditional occupational therapy.

This article offers a comprehensive review of robotic systems in the assessment and treatment of upper extremity disorders and impairments. The review also goes deeper than most of the literature by exploring how these robots are created, how popular motor learning principles operate, and how they could be applied, as well as exploring some of the clinical trials and their results. The main contribution of this paper is to conduct a comprehensive review of the advancements in robotic systems to treat upper extremity disorders and impairments. The main theme of the paper is to review what has been achieved so far in this field and discuss the gaps and the challenges that need to be addressed to achieve higher dependence on robotic systems to effectively deal with rehabilitation systems

The rest of the paper is organized as follows. Section 2 provides a detailed classification of various robots used in rehabilitation. Different strategies to control rehabilitative robots are discussed in Section 3. Motor learning strategies are discussed in Section 4. Section 5 illustrates some experimental clinical trials, while Section 6 presents the conclusion.

## 2. Classification of Rehabilitative Robots

**Figure 1:**
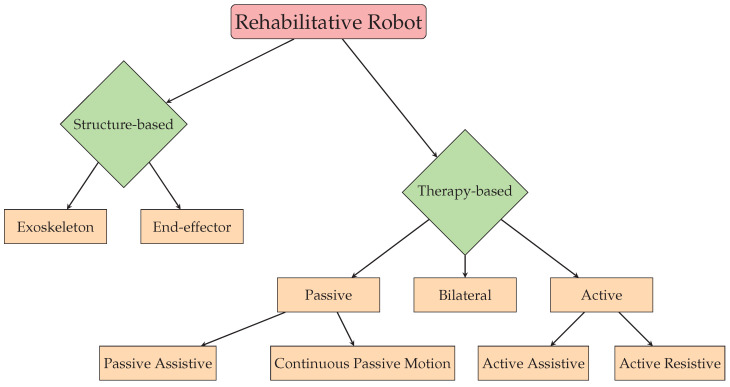
Classification diagram.

Rehabilitative robots can be classified in two ways, i.e., according to their structure and according to the type of provided therapy. Figure 1 shows the classification tree.

### 2.1. Structure-Based Classification

Rehabilitative robots can be classified in similar ways to any other robots [9]. In this section, they are classified based on their mechanical structure. The two types of structure-based classifications for rehabilitation robots are exoskeletons and End-Effector (EE)-based systems.

#### 2.1.1. End-Effector-Based Systems

**Figure 2:**
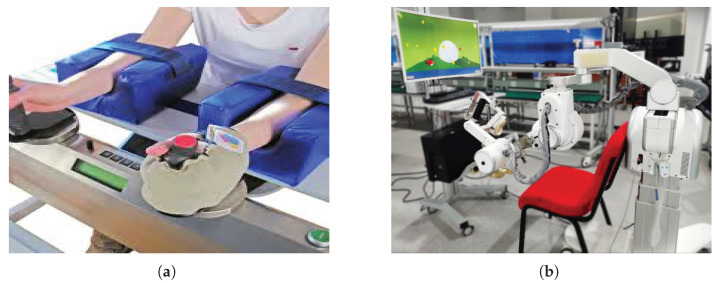
Structure-based classification. (**a**) Bi-Manu-Track end-effector-based robot (2013). (**b**) The FLEXO-arm1 exoskeleton (2021).

The earliest literature on robotic UE therapy was using EE-based systems. These systems only have one interface, the hand/forearm of the patient. This type of system is simpler and easier to manufacture; moreover, they are more flexibly adjustable to fit different arm lengths, although determining the patient’s posture proves difficult with this type of system. It also disallows generating isolated movements at specific joints, since movement at the EE can cause a combination of movements in the entire limb’s joints (wrist, elbow, and shoulders) [10]. Examples of EE-based systems include:

- MIT-MANUS, a system with five degrees of freedom (DOF) that helps in the rehabilitation of three degrees of freedom of wrist motion: extension-flexion, abduction-adduction, and pronation-supination [11,12];
- Bi-Manu-Track (Figure 2a), a single DOF robot that supports pronation and supination for the forearm and the wrist [13];
- Mirror-Image Motion enabler (MIME), a six-DoF robot that is used for the shoulder and elbow [14,15].

More examples of EE-based systems are included in Table 1.

#### 2.1.2. Exoskeletons

**Figure 3:**
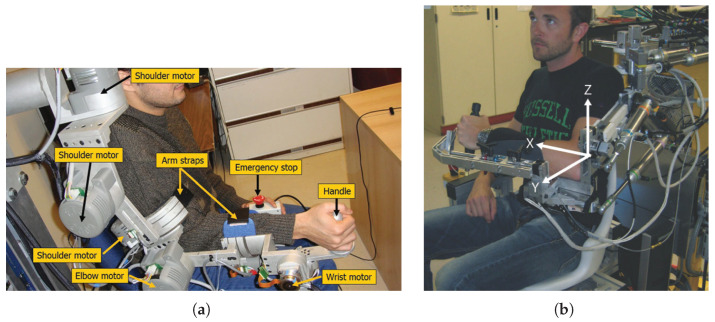
Passive assistive devices. (**a**) ETS-MARSE. (**b**) PNEU-WREX.

Exoskeleton robots resemble human limbs and are connected to patients at multiple points, where their joint axes match with human joint axes. As such, they provide more accurate joint isolation and increase gait transparency. They also allow the training of focused muscles as controlling specific joint movements is possible. Examples of exoskeleton robots include:

- FLEXO-arm1 (Figure 2b): The FLEXO-arm1 was developed collaboratively by the Shanghai Engineering Research Center of Assistive Devices and the University of Shanghai for Science and Technology [16].
- Harmony: The exoskeleton known as Harmony is equipped with a shoulder mechanism that follows the natural anatomy of the human body, allowing for unrestricted movement of all joints. It is capable of bearing the weight of the upper body and applying assisting force to help patients carry out desired movements [17].
- ANYexo: The ANYexo exoskeleton is a flexible and adaptable device with six degrees of freedom, intended for use on the upper limb. It is equipped with a series of elastic actuators that allow for low-impedance torque control. The device is primarily used as an experimental platform to test new hardware concepts and algorithms for autonomous therapy of patients with varying degrees of neural impairment. The aim of the device is to provide greater independence and functionality to individuals with arm impairments [18].
- ETS-MARSE (Figure 3a): The ETS-MARSE is a seven-DoF robot that consists of a shoulder motion support part, an elbow and forearm motion support part, and a wrist motion support part [19].
- MyoPro: (https://myomo.com/what-is-a-myopro-orthosis/, accessed on 25 April 2023) MyoPro is a powered upper limb orthosis developed by Myomo Inc. (Boston, MA, USA). The device is designed to help individuals with upper limb paralysis due to conditions such as stroke, spinal cord injury, or brachial plexus injury regain movement and function in their affected arm.
- Neofect Rapael Smart Glove: (https://www.neofect.com/us/smart-glove, accessed on 25 April 2023) Neofect Rapael Smart Glove is a wearable glove that uses sensors and haptic feedback to provide interactive training for individuals with hand weakness due to neurological conditions. The device is designed to help patients regain fine motor control and dexterity in their hands.

**Table 1:** Comparison between exoskeletons and end-effector-based systems.

	End-Effector-Based Systems	Exoskeletons
Advantages	Faster to set up	Increased gait transparency
	Easier to manufacture	Isolated joint control
Disadvantages	Less sophisticated	Expensive
	Limited joint control	Not easily adjustable to different arm lengths
Examples	[11,12,13,14,15,20,21,22,23,24,25,26,27,28,29,30,31,32,33,34,35,36,37,38]	[19,39,40,41,42,43,44,45,46,47,48,49,50,51,52,53,54,55,56,57,58,59,60,61,62,63,64,65,66,67,68,69,70,71,72,73,74,75,76,77,78,79,80,81,82,83,84,85,86,87]

Home-based end-effector neuro-rehabilitation systems are an emerging area of research and development that holds promise for improving access to therapy for individuals with neurological impairments. These systems typically consist of a robotic device that is attached to the distal end of the affected limb and provides assistance or resistance to movement during therapy sessions. One advantage of home-based systems is that they can provide more frequent and intensive therapy than is typically available in a clinical setting, which may lead to better outcomes. Additionally, home-based systems can be more convenient and cost-effective for patients, who may have difficulty traveling to a rehabilitation center or who may not have access to specialized therapy services in their area. Recent studies have shown the potential effectiveness of home-based end-effector neuro-rehabilitation systems, such as the pilot study by Bressi et al. [88]. The study aimed to investigate the effects of a robotic home-based treatment rehabilitation using the iCONE robotic device on stroke patients with chronic conditions and without the presence of a therapist during exercise. Patients underwent an initial and final assessment, followed by 10 days of at-home treatment. Results revealed significant improvements in robot-evaluated indices and patient satisfaction, indicating the potential of the robotic rehabilitation approach in reducing healthcare costs, ensuring continuity of care, and reaching patients in distant or resource-limited areas.

Another home-based end-effector neuro-rehabilitation system is presented in [89]. This research aims to develop a telerehabilitation framework that can remotely provide home-based passive rehabilitation therapies to individuals with upper limb dysfunctions using a desktop-mounted rehabilitation robot (DMRbot) and PTC’s Industrial Internet of Things (IIoT) platform. An experiment was conducted with two healthy male human subjects, where the rehab robot data and therapists’ commands were transported by ThingWorx to evaluate the ability of the therapist to provide telerehabilitation and the device’s performance. The results showed that the proposed end-effector type therapeutic robot for home-based upper limb rehabilitation resulted in 100% trajectory movement of the patient’s hand, with a network latency of approximately 0.15 s, indicating that the proposed framework can make therapies more approachable to remote areas with convenience and affordability. Although promising results were obtained with home-based end-effector neuro-rehabilitation systems, further research is needed to fully evaluate the feasibility, safety, and efficacy of home-based end-effector systems and to identify the optimal design features and protocols for home-based therapy.

### 2.2. Therapy-Based Classification

It is important to choose the appropriate type of therapy for the patient at hand [90], as it influences the effect of the treatment to a great extent. There are three types of therapy, i.e., passive, active, and bilateral [91]. Passive therapy is when the patient does not have to exert any force or effort to complete the exercise. In occupational therapy, this is done when the therapist moves the patient’s afflicted limb to do a specific motion. This type of therapy is usually utilized in the early stages of post-stroke symptoms when the impaired limb does not respond [92]. On the other hand, patients who can move their damaged limb to a certain extent are prescribed active therapy, which is when a patient at least attempts the exercise with assistance as needed from the therapist. Finally, there is bilateral therapy, which is used when the patient can move one but not both of their arms, doing a mirror-like exercise where the impaired limb is copying the functional limb. Table 2 illustrates a brief comparison between therapy types.

#### 2.2.1. Passive Therapy

In the context of rehabilitative robots, passive therapy is where there is no input provided from the patient. As such, passive robots are further divided into two categories, i.e., CPM and passive assistive robots.

CPM: In Continuous Passive Motion, a machine is used to move the joint without the patient having to exert any effort. The motorized device gently bends the joint back and forth to some degree to complete preprogrammed motions without any interference from the patient. One such CPM device is the ETS-MARSE (Figure 3a) [19], which is an exoskeleton capable of performing both passive and active therapy. In its passive operation mode, the ETS-MARSE moves the user’s arm to a preprogrammed trajectory.

Passive Assist: Unlike CPM robots, passive assistive devices do not move in preprogrammed trajectories; however, being passive devices, their movement does not depend on any control input. These usually come in the form of gravity compensation, such as the T-WREX [83], the non-robotic actuated version of the PNEU-WREX (Figure 3b) [46], both of which give the patient a sense of floating, making it easier for the patient to move their arms.

#### 2.2.2. Active Therapy

As the antithesis of passive devices, active devices read input from the patient using them to determine how the device should move, whether assistive or resistive motion.

**Figure 4:**
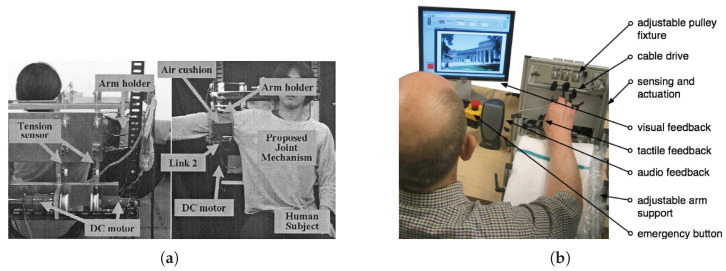
(**a**) Active Assistive: Kiguchi et al. uses a neuro-fuzzy controller and controls *admittance* [76]. (**b**) Active Resistive: *HandCARE*, in its resistive mode of operation, resists motion by controlling *impedance* [31].

Active Assistive: Active Assistive devices are similar to Passive Assistive devices in that they both assist the user to complete the motion. However, active robots use some sort of sensor to detect the patient’s motor activity, such as the surface Electro-Myography (sEMG) sensor implemented in Kiguchi et al. (Figure 4a) [76], which uses a neuro-fuzzy controller to determine the user’s intention of shoulder motion and uses a perception assistance control strategy. On the other hand, the force/torque sensors are used for admittance control to determine how fast the robot should move or stop.

Active Resistive: Active resistive devices follow the same control principles as active assistive devices, since they are controlled based on some sort of input from the user. However, instead of promoting skill acquisition similar to active assistive devices, they promote motor adaptation [93]. Similar to a force field, active resistive devices make completing the movement harder, introducing a perturbation to which the brain can adapt. *HandCARE* (Figure 4b) is an EE-based system with five DoF for the independent linear movement of each finger. It has both modes of active therapy, which use the position of the fingers for control of assistance/resistance. In the case of resistance, that is done through impedance control [31].

#### 2.2.3. Bilateral

Bilateral therapy is performed by mirroring the motion of the patient’s arm, and the mirrored arm is used to complete the exercise. This is done by the robot, and the patient is not involved [94]. Bilateral therapy first came into existence with the development of the Mirror-Image Motion Enabler, or MIME for short [15]. Table 3 and Table 4 give a summary of the robotic rehabilitation systems based on the therapy type, while Table 5 discusses the advantages and disadvantages of known rehabilitation robots.

## 3. Control Strategies

**Figure 5:**
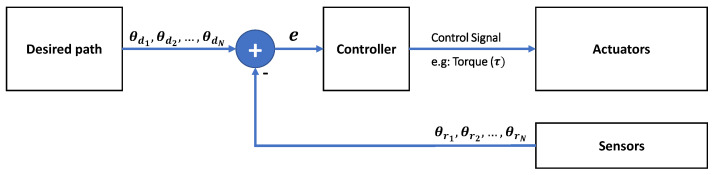
General illustration of the different components of a robotic system being controlled using a feedback control system.

This section explores different control strategies applied within a robotic upper extremity rehabilitation system. A control system is responsible for ensuring the stability of the robotic system by calculating the forces needed to follow a pre-defined path. Figure 5 shows a general illustration of a feedback control system applied to a robotic system.

The three components that build a robotic system are the controller, the actuators, and the sensors. The sensors are the devices that measure signals in the environment. The controller processes the data from the sensors, and in turn, uses the data to control the actuators. As stated in the previous section, passive devices do not require input to the controller. However, when speaking in terms of active and bilateral devices, controllers implemented in the literature have employed the use of numerous signals to determine how the actuators should move.

### 3.1. Controller Input

The sensor and the processor work together in what is called impedance control, where the computer uses the data gained from the sensor to determine how much “action” the effectors should perform. Some exciting ways were implemented to achieve impedance control. We shall start off with what is probably the most unique: perception assistance. The idea of perception assistance is to read EMG signals, or in the case of [68], EMG signals in combination with EEG signals, to predict the patient’s intention of movement and supplement it accordingly. This method was proposed with the MIT-MANUS [11,105], the very first robotic device deployed in clinical trials delivering rehabilitation therapy [106], where EMG signals were to be collected from different muscles on the shoulder and elbow. The assistance is triggered when the EMG activation amplitude exceeds a threshold (similar to an on/off controller). The MIT-MANUS does not use that, however; instead, it uses joint positions, angular velocity, and torque as its control signals [107]. Other devices, however, rather than use the EMG signals as on/off controllers, as what was proposed with MIT-MANUS, generate assisting forces proportional to the EMG signal. This was achieved in [76], where Kiguchi et al. passed the EMG signals to a neuro-fuzzy controller, artificial neural networks (ANN), and fuzzy logic to accurately determine the impedance of the exoskeleton’s degrees of freedom. Moreover, as stated earlier, a study was conducted where EEG signals were used in combination with sEMG signals. That study tested the viability of using EEG signals in an existing exoskeleton, the SUEFUL-7 [63], and in a later study, this technique was found to be better than sEMG signals alone, achieving an average hit rate of 84.0% and average miss-hit rate 30.0%, compared to average hit rate 79.7% and average miss-hit rate of 31.6% when using EMG signals alone [108]. Other systems [64] have used pneumatic actuators. The impedance was controlled by using the cylinder pressure as the control input. Generally, for an upper extremity robot, the force/torque mapping from cylinder coordinates to shoulder joint coordinate can be expressed as follows:

(1) $$\tau =J^{T}C$$

where τ is the joint torque vector, *C* is a 4D vector of cylinder forces, and $J^{T}$ is the transpose Jacobian matrix of the mapping for a given configuration. The majority of the systems, however, use simpler inputs, such as force/torque of the patient’s movement and EE positions. Table 6 offers a list of control signals and the references that use them.

### 3.2. Actuation

Along with the sensors, there is a multitude of actuators that can be chosen to control the system. These include but are not limited to electric (AC/DC) motors and hydraulic and pneumatic actuators. Some robots, such as the T-WREX [83] do not have any robotic actuators. Analogous to the EMG sensors that are used for sensing the patient’s intention of movement, there are Functional Electric Stimulation (FES) [109] actuators which are used to control the patient’s movement by sending low-level electrical impulses to the nerves within the muscle. FES differs from other actuators in that it is not used to control an effector, but instead, the signal is sent to *the human* user of the robotic device. As with the previous section, Table 7 includes a list of actuators and some of the robotic devices that use them.

### 3.3. Controllers

This section examines the different controller designs which were implemented in an upper-extremity rehabilitative robotic system. Several control paradigms were utilized in rehabilitative robotic systems ranging from basic controllers such as the Proportional-Integral-Derivative controller (PID) to hybrid complex controller designs. The variety of the available controller designs gave the developers the flexibility to choose the appropriate controller based on the advantages and disadvantages of each control class. In the literature, the controllers of an upper-extremity rehabilitation robotic system can be classified into PID controllers, robust controllers, adaptive controllers, and Artificial Intelligence (AI) controllers. Table 8 gives a summary of the robotic rehabilitation systems based on the controller type. Table 9 shows the advantages and disadvantages of each control strategy.

#### 3.3.1. PID Controllers

One of the most simple and basic controllers is the PID controller. A PID controller continuously applies a command signal to the actuators to minimize the error between the desired values and the sensed actual values based on proportional, integral, and derivative terms. The simple design and philosophy of PID controllers have been utilized successfully in many different robotic applications with an acceptable accuracy degree. In [110], the Firefly Algorithm (FA) is used to optimize the PID parameters for the upper extremity rehabilitation robot. The robotic system was an exoskeleton of a three-DoF system designed to facilitate the movements of the elbow and the shoulder. Another PID controller proposed for upper extremity rehabilitation is presented in [111]. The simulated annealing algorithm was used to optimize two Proportional-Derivative (PD) controller gain sets. The robot was a musculoskeletal system based on a five-DoF arm model and 22 muscles. The parameters of the controller were optimized by minimizing a weighted sum of the position, orientation, and muscle activation errors. In [113], a linear PID controller is designed for the exoskeleton system (EXO-UL7). The work claimed to achieve asymptotic stability and proved the effectiveness of the proposed system in simulation.

PID controllers have been widely implemented in commercial-level products. The main advantage of the PID controller is the simplicity of the design. The PID simply consists of a single equation and does not need any knowledge of the plant to be controlled. Acceptable performance can be achieved when operating in normal conditions, which is usually the case with rehabilitation robots. Rehabilitation robots are usually operating in stable indoor environments with minimal external disturbances from the environment. Therefore, as presented earlier, the literature shows a number of successful rehabilitation robotic systems have adopted PID controllers. However, it is clear from the presented works that the PID itself was not enough to produce advanced performance, and thus, an additional optimization step was needed to find the best tuning parameters of the PID controller.

#### 3.3.2. Robust Controllers

Even though the successful integration of PID controllers in rehabilitation robots, the performance of PID controllers cannot be always guaranteed. If the system is exposed to a sudden strong disturbance from the environment, the PID controller might not be able to handle and maintain the stability of the robotic system. Moreover, rehabilitation robotic systems are directly connected to the body of a human. A small wrong movement can cause critical injuries. Therefore, to ensure better performance and to achieve advanced abilities to reject disturbances, other classes of controllers are adopted in rehabilitative robots, such as robust controllers, adaptive controllers, and AI-based controllers.

**Figure 6:**
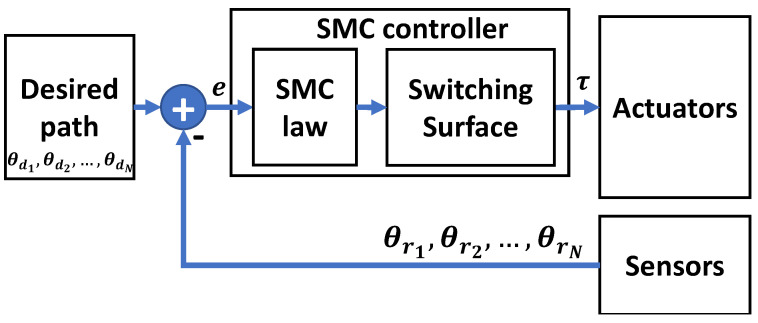
A block diagram of a general SMC controller design.

The robust control theory focuses on dealing explicitly with uncertainties and disturbances that are defined within a pre-defined boundary. Robust controllers achieve stability in the presence of bounded modeling errors. A well-known robust control technique is the Sliding Mode Control (SMC). Figure 6 shows a general block diagram of SMC control design. An SMC controller commands the system states towards a suitably designed desired surface, known as the sliding surface. With the assistance of a properly designed control law, the system states should remain on the surface.

It is known that SMC controllers can achieve more robust results compared to basic PID controllers. Therefore, the literature has shown a number of rehabilitative robotic systems controlled using SMC controllers. In [114], a fractional sliding mode control (FSMC) is utilized to control their proposed u-Rob robot, which is a seven-DoF exoskeleton that features shoulder scapulohumeral rhythm with a wide range of motions. The stability of the proposed FSMC controller was examined using Lyapunov theory. Moreover, the proposed FSMC was able to deal effectively with unmodeled dynamics, such as friction and disturbances. The proposed FSMC was proved to achieve better tracking and enhanced chatter behavior compared to basic SMC controllers. Another SMC work proposed to control an upper rehabilitation robot is proposed in [115]. The system is a fuzzy sliding mode control named NFSMC which is proposed to control a seven-DoF upper-limb exoskeleton robot. First, a novel sliding surface is designed to be able to overcome external disturbances and unknown dynamics, including friction, input saturation, and various upper limb masses. Based on the simulation results, the proposed NFSMC technique was able to achieve high trajectory tracking. In [116], a new exoskeleton robot is designed and controlled for shoulder rehabilitation. The measurements of the robot are similar to the properties of the upper limb of an adult. For the third joint, a novel open circular mechanism is adopted. The study has also presented the forward and inverse kinematics of the proposed robot, the singular points, the Jacobian matrix, and the dynamics of the robot. After that, an SMC controller is proposed to achieve the desired trajectory. The reason for favoring SMC controllers over conventional PID controllers in this work is to be able to control the robotic system with an advanced robustness level and higher abilities to resist uncertainties, parameter changes, and disturbances applying to the system, such as a patient’s hand tremor. The SMC parameters were optimized using the Genetic Algorithm (GA). The proposed exoskeleton is a low-weight robot with a special mechanism for the third joint. This proposed design allows translational degrees of freedom of the shoulder, which makes the proposed robot comfortable for the patient.

#### 3.3.3. Adaptive Controllers

**Figure 7:**
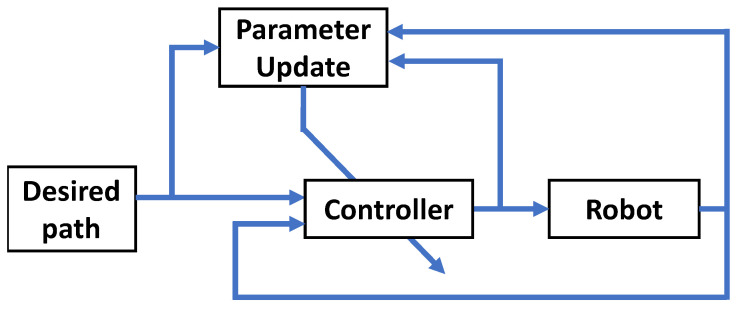
A block diagram of a general design of adaptive controllers.

The third class of controllers used in rehabilitation robots is the adaptive control method. Figure 7 shows a general block diagram of adaptive controllers. This control strategy adopts a controller which changes its behavior to adapt to the changes in the plant parameters or to uncertain parameters. The difference between the robust control strategy and the adaptive control strategy is that the adaptive control strategy does not need prior information on the boundary of the uncertain parameters of the system. A parameter estimator provides updated laws to automatically modify estimates based on the sensed data. A well-known adaptive-based controller is the Active Disturbance Rejection Control (ADRC) method. The rationale of the ADRC method is to create a fictitious state that includes all possible uncertainties and disturbances. An online estimation of the disturbances is performed using an Extended State Observer (ESO), which is then fed back to design a suitable controller to detach the system from all uncertainties and disturbances. Compared to PID controllers, ADRC controllers share the advantage of simplicity. However, ADRC controllers have advanced abilities to reject disturbances and uncertainties.

Thus, several recent rehabilitation robots have been proposed to be controlled by an ADRC controller. In [117], an ADRC controller was implemented for the application of governing a proper realization of limb rehabilitation exercises. The proposed ADRC technique was implemented on a flexible joint arm model, which is similar to a real rehabilitation robot. The modeling and control of such systems are challenging due to the multidimensional character of the assisting mechanism. Therefore, the ADRC controller is adopted to decouple the uncertainty from the system. This has enhanced the robustness of the robotic system against disturbances. Another ADRC-based control is proposed in [118]. This work proposed a non-linear ADRC method composed of a non-linear ESO and a non-linear state error feedback to track a pre-defined trajectory. This non-linear ADRC controller is implemented in an upper limb exoskeleton consisting of two links. The links can mimic flexion/extension movements for both the elbow and the shoulder. The dynamic model of the exoskeleton was developed using the Euler–Lagrange method. The robustness of the proposed control strategy was tested when applying four disturbance cases with 20% parameter variation. The simulations have shown that the proposed ADRC controller was able to reject disturbances and achieve advanced tracking results compared to PID controllers and even over other conventional ADRC methods. For the same application of [118], a different controller based on ESO and a finite time stable tracking differentiator (FTSTD) is proposed that showed superior performance over non-linear ARDC [119]. In [120], and a novel rehabilitation robot to assist therapists performing repetitive hand group stretching was also proposed. The actuator of the proposed system is based on elastomeric materials. Thus, the proposed robot is softer and lighter than classical rigid exoskeletons which are used for hand rehabilitation. Building an exact model of such objects is not a trivial task. Therefore, an ADRC technique combined with an ESO is proposed to deal with the uncertain model of the robot. The proposed technique showed superior transient and steady-state performances and better disturbance resistance ability compared to the PID control strategy. Moreover, the Reduced-order Extended State Observer (RESO) was also studied. The proposed device was experimentally tested and proved the effectiveness of the system in several clinical tests.

#### 3.3.4. AI-Based Controllers

**Figure 8:**
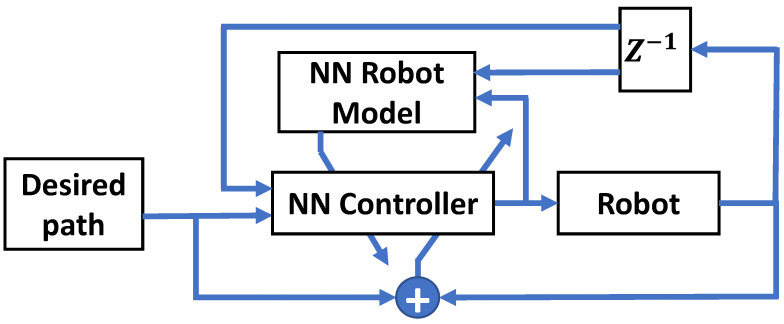
A block diagram of a general design of NN controllers.

With the recent advancements in the processing power of modern computers, Artificial Intelligence (AI) concepts have been widely adopted in control theory. A popular AI-based tool is Neural Networks (NN). Figure 8 shows a general design of an NN-based control system. NN-based controllers are gaining increased interest within the few last years due to their high ability to operate with “black-box” systems. Even with the absence of a model of the plant, NN-based controllers can obtain the model of the plant by observing the plant output when changing the input values and develop a relationship between the input and output. Thus, NN-based controllers are preferable in the cases where the plant is difficult to model.

In [121], a novel impedance controller based on Evolutionary Dynamic Fuzzy Neural Network (EDRFNN) is proposed. The required impedance between the impaired limb and the rehabilitation robot is regulated in real time based on the physical condition of the impaired limb. First, an online estimation of the stiffness and the damping parameters of the impaired limbs is performed using a Slide Average Least Squares (SALS) technique. After that, the Genetic Algorithm (GA), dynamic Back-Propagation (BP), and Hybrid Evolutionary Programming (HEP) algorithms are adopted as the learning algorithms for the EDRFNN impedance controller. The GA and HEP are utilized to optimize DRFNN parameters to obtain semi-optimal parameters. Based on the error gradient descent technique, the Dynamic BP learning method is fine-tuned in a real-time manner. The stability of the proposed system was proven using the discrete-type Lyapunov function. Simulations have shown that the proposed control system can achieve acceptable performance even when altering the condition of the impaired limb. Another work based on NN concepts is proposed in [122]. The proposed work is an adaptive admittance control system based on an NN-based disturbance observer built to control a rehabilitation robot that assists patients in moving their upper body parts. The disturbance observer NN structure is a radial basis function network that is responsible to deal with the uncertainties and modeling errors. Three volunteers have tested the proposed system with several experiments, such as tracking some desired paths including sinusoidal and circular paths with resistive training experiments. The experiments have proven the effectiveness and robustness of the proposed control system, which was able to provide patient-passive and patient-cooperative rehabilitation training.

## 4. Motor Learning Strategies

While the control methods are the engineer’s point of view, rehabilitative robotics are a multidisciplinary study that no one short of a polymath can do on their own, and medical personnel’s input is a must for a successful system. This section aims to provide the strategies targeted at the human brain, rather than the robotic devices, to induce motor learning. They are inevitably parallel, and as such, there are many similarities that a bijection is *almost* possible between the two perspectives.

### 4.1. Game Therapy

**Figure 9:**
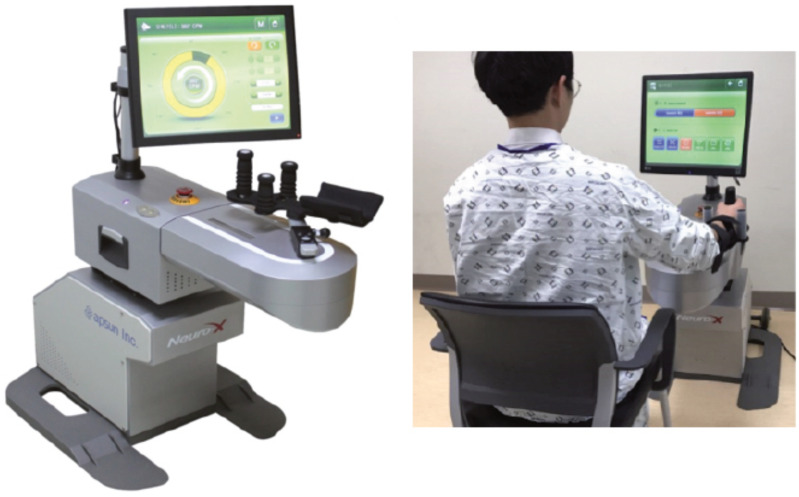
Neuro-X (Apsun Inc., Seoul, Korea).

The study in [93] delved into the motor learning principles for neurorehabilitation, where they discussed the difference between motor adaptation and skill learning. Two distinct types of motor learning were considered: adaptation and skill acquisition. Adaptation refers to the response to a perturbed environment to regain a previous level of performance [123]. Conversely, skill learning involves learning new patterns of muscle activation [124,125,126]. The clear difference between these two principles is implicitness and explicitness. Implicit learning refers to acquiring skills without awareness or directing them to a conscious level. This contrasts with explicit learning, which refers to the acquisition of a skill that one directs or another directs [127]. With that said, it was found that the implicit training paradigm could lead to a greater learning effect than that of the explicit model [128,129]. One way researchers achieved implicit learning in robotic systems in the literature is by utilizing Game Therapy. The T-WREX and the PNEU-WREX are both supplemented with the Java Therapy program. Java Therapy has a library of evaluation and therapy activities that can be played with a commercial force feedback joystick as well as the robotic systems mentioned earlier [130]. The MIT-MANUS [11] and the MIME [15] also follow such a strategy. Other systems that involve game therapy include [26,38,39,43,53,60,86]. In a randomized controlled trial, game therapy was tested against conventional therapy. The results conducted that game therapy is not superior to conventional therapy, and can only act as an additional therapy to increase the amount of rehabilitation. However, it was found that it may be more effective if game therapy is started before 30 days of the stroke incident [131]. In [132,133], the same was tested with robotic assistance using the upper extremity rehabilitation robot Neuro-X (Apsun Inc., Seoul, Korea) (Figure 9). Although the authors have declared that the study was limited and additional research is needed, the verdict was that there was no significant difference between the groups. The authors do however indicate that robot-assisted game training induces patients to perform accurate motions in a more psychologically non-quantifiable way.

### 4.2. Virtual Reality

**Figure 10:**
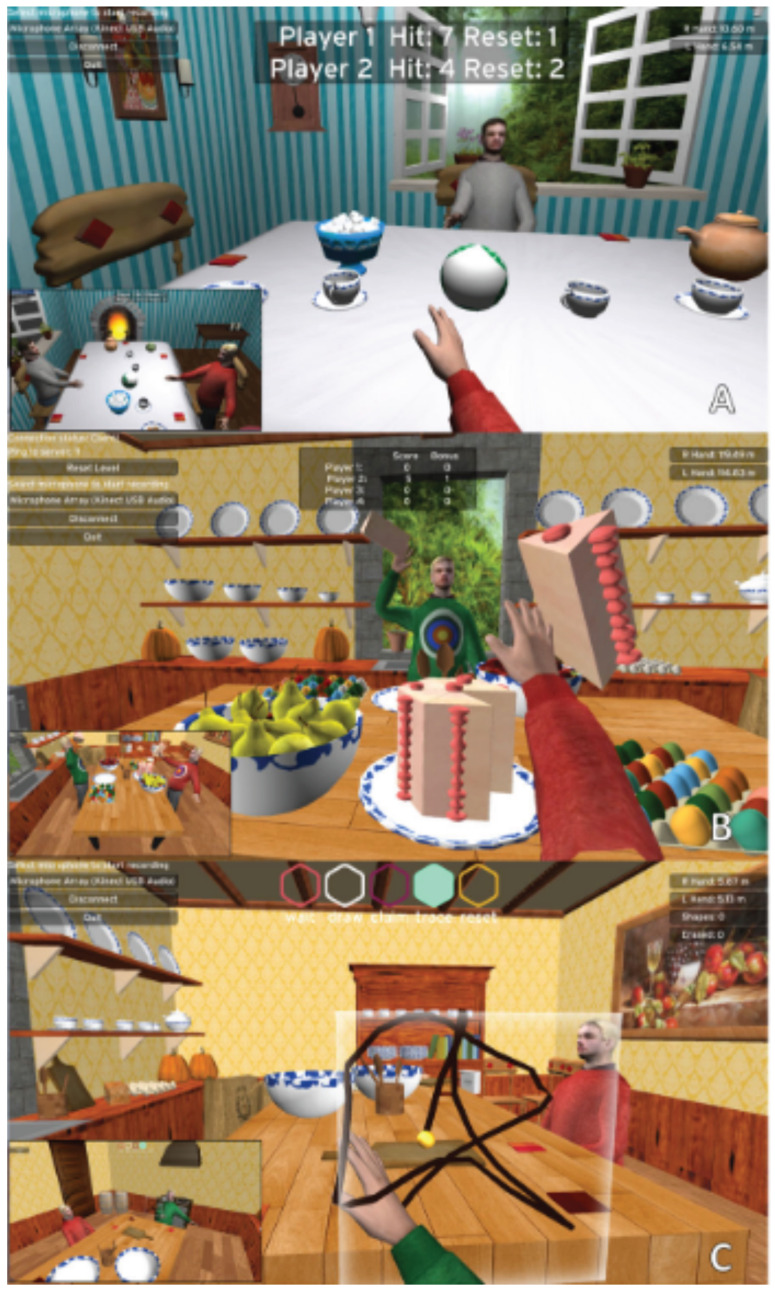
The VERGE Virtual Reality System [136]. (**A**) Ball bump. (**B**) Food fight. (**C**) Trajectory Trace.

Game therapy also takes on a more immersive form by utilizing Virtual Reality (VR) technology. Previously, VR was limited by the technologies of the time, but as development advanced, VR technologies have become more accessible, which quickly revealed their potential in medical use cases [134]. In the context of upper limb rehabilitation, users may interact with virtual objects that mimic the real world, with feedback ranging from visual queues on the headset to haptic feedback. Levin et al. [135] described how this technology can be correctly implemented in upper extremity rehabilitation, and how motor learning paradigms should be implemented in a virtual setting. In a randomized trial, multiuser VR therapy was compared to single-user VR therapy, both home-based trials. After four weeks (two weeks of the single user version and two weeks with the multiuser version) of in-home training using a multiuser VR system called VERGE (Figure 10) [136], the FMA score improved significantly across all participants [137].

Unfortunately, a Cochrane review [138] showed that VR may not be more effective than conventional therapy.

## 5. Clinical Trials

This section aims to showcase a number of randomized trials that were carried out over the years. We will first cover some of the popular and quantifiable motor ability tests in the following section, and then some of the trials in Section 5.2

### 5.1. Quantifying Motor Recovery

In order to assess the effectiveness of the devices used, the effect sizes must be defined. There exists a number of ways to assess motor recovery that are attested by clinicians, physiotherapists, and researchers. The following are some of the most common ways to assess motor recovery, which can be used to understand the results of RCTs and meta-analyses, as they measure different goals.

#### 5.1.1. Fugl-Meyer Assessment

The Fugl-Meyer [139] scale is a 226-point multipoint Likert scale that was developed as an assessment measure of recovery from a hemiplegic stroke. This assessment is used in both clinical settings and research settings to evaluate the aforementioned recovery. It has been described by Gladstone et al. [140] as “a much-needed instrument for monitoring the course of recovery from hemiplegic stroke”. It encompasses five areas: motor function, sensory function, balance, joint range of motion, and joint pain. Each area contains several items, each of which is rated on a three-point ordinal scale (0 = no performance, 1 = partial performance, 2 = complete performance). The motor domain includes items that measure movement, coordination, and reflexes in the shoulder, elbow, forearm, wrist, hand, hip, knee, and ankle. The motor score ranges from 0 (hemiplegia) to a maximum of 100 points (normal motor performance), divided into 66 points for the upper limb and 34 points for the lower limb. There is also a maximum of 24 points for sensitivity, 14 points for sitting and standing balance, 44 points for joint mobility, and 44 points for joint pain. It is considered by many in the field of stroke rehabilitation to be one of the most comprehensive quantitative measures for motor impairment after stroke and is recommended to be used in clinical rehabilitation studies. This scale is an objective disease-specific deterioration index that was specially developed as an assessment measure to assess recovery in patients with hemiplegia after stroke. The upper extremity section of the FMA (FMA-UE) is what we are concerned about within this review, and is scored out of 66.

#### 5.1.2. Action Research Arm Test

The action research arm test (ARAT) [141] is a 19-item observational method used by physical therapists and other health care professionals to evaluate upper extremity performance (coordination, dexterity, and function) in stroke, brain injury, and multiple sclerosis recovery groups. ARAT was originally described by Lyle in 1981 as a modified version of the upper extremity function test and was used to study the recovery of upper extremity function after cortical injury. The elements that make up the ARAT are categorized into four sub-levels (grip, grasp, pinch, and gross movement), ranked in order of decreasing difficulty, with the hardest task considered first, followed by the least difficult task. Lyle suggested that this pecking order would improve inspection efficiency, as normal movement on harder items would be indicative of successful performance on current items. task performance is rated on a four-point scale, from 0 (no movement) to 3 (normally performed motion).

#### 5.1.3. Wolf Motor Function Test

**Figure 11:**
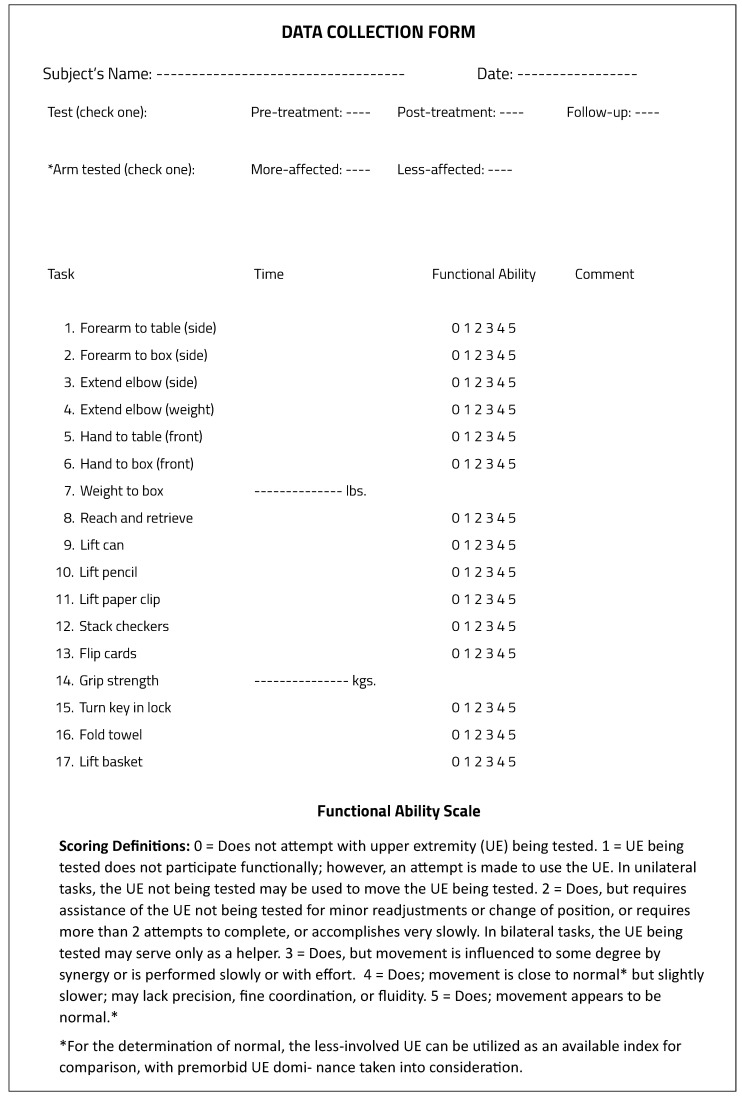
The data collection form for the WMFT [143].

The Wolf Motor Function Test (WMFT) quantifies UE motor ability through timed and functional tasks [142]. The widely used version consists of 17 tasks, 15 function-based tasks, and 2 strength-based tasks. The data collection form, including the scoring information of WMFT is shown in Figure 11 [143].

#### 5.1.4. Stroke Impact Scale

Another scale that is used to assess motor recovery is the stroke impact scale. It is a stroke-specific self-reporting and health measure. It is designed to evaluate multidimensional stroke outcomes. The scale was first released as version 2.0, which includes 64 items in 8 domains (strength, hand function, activities of daily living (ADL)/instrumental ADL, mobility, communication, emotion, memory and thinking, and participation) [144]. Based on the results of the Rasch analysis, 5 items were removed (with 59 items remaining after the removal) from version 2.0 to create the current version3.0 [145]. Each item is rated using a five-point Likert scale, where 1 is an inability to complete the item and 5 is no difficulty experienced at all.

#### 5.1.5. Barthel’s ADL Index

Barthel’s ADL index [146] is an ordinal scale used to measure performance in ADL activities. Ten variables describing ADL and mobility are scored: 0 being “in content”, 1 being “occasional accident (once/week)” and 2 being “content”. The Barthel index measures the degree of assistance required by an individual on 10 items of mobility and self-care ADL. Thus, overall scoring ranges between 0 and 20, a higher number being a reflection of greater ability to function independently following hospital discharge [147].

### 5.2. Trials

Table 10 summarizes the robotic rehabilitation clinical trails attempts. The first clinical trials employing rehabilitation robots looked at whether they might be useful compared to traditional treatment. The MIT-MANUS provides therapy that uses repetitive massed practice of reaching toward an endpoint. In terms of pain, no difference was found between the groups, with seven controls and five experimental subjects developing pain in joints or tendons, and of these, three controls and four experimental subjects developed the shoulder-hand syndrome. Additionally, there were no adverse events in the estimated 500 h of operation, and it was well-received by the patients [12]. In another study [148], the therapists expressed a qualified acceptance. Clinical values were also reported in [12]. However, there exists a much more recent, randomized controlled trial, dubbed RATULS (Robot-Assisted Training for the Upper Limb after Stroke), which was carried out at four UK centers [149]. RATULS had 770 participants enrolled in 3 groups, where 257 were assigned to robot-assisted training, 259 were assigned to enhanced upper limb therapy (EULT), and 254 were assigned to usual care. FMA for the robot training group was 68.9 (16.5) at baseline, 76.6 (22.1) after 3 months, and 78.2 (22.8) at 6 months. The FMA of the EULT group was 69.0 (17.9) at baseline, 77.8 (22.8) after 3 months, and 79.4 (24.1) at 6 months. The FMA of the usual care group’s was 68.9 (17.4) at baseline, 74.2 (23.6) after 3 months, and 77.9 (23.2) at 6 months.

The ARMin III was tested in a parallel-group randomized trial of 24 sessions [154]. The results show that the FMA-UE score of the robotic treatment group was 2.6, 3.4, 3.4, and 3.1 at 4, 8, 16, and 34 weeks, respectively. Compared to the control group’s results, which were 2.0, 2.6, 2.8, and 2.9 at 4, 8, 16, and 34 weeks, respectively, it shows a significantly greater improvement for the robotic treatment group, but the gap between the two groups was closer as time went on.

A single-blinded randomized trial was carried out with the Bi-Manu-Track, where 44 patients were split into a control group and a robot arm trainer (AT) group, who participated in 30 sessions in either conventional therapy (control group) or robotic therapy using the Bi-Manu-Track (AT group). In the robot AT group, FM score was 15 points higher at study end and 13 points higher at 3-month follow-up than the control ES group [152].

The T-WREX also had a randomized controlled trial that had an intervention carried over 24 Sessions. The robotic treatment group saw an FM improvement of 3.3±2.4 (p=0.001) and 3.6±3.9 after a 6-month follow-up (p=0.005) compared to control group’s FM improvement of 2.2±2.6 (p=0.004) and 1.5±2.7 after a 6-month follow-up (p=0.06) [150]. The other robot, the PNEU-WREX, has been tested in a Randomized Controlled Trial, where 26 subjects were split into two groups and partook in 24 sessions across a 2-month period. The group that received the robotic training saw an FM improvement of 3.0±4.9 (p=0.02) and 2.4±5.2 after a 3-month follow-up (p=0.06) compared to control group’s FM improvement of 0.9±1.7 (p=0.04) and 0.1±1.4 after the 3-month follow-up (p=0.4) [153].

**Figure 12:**
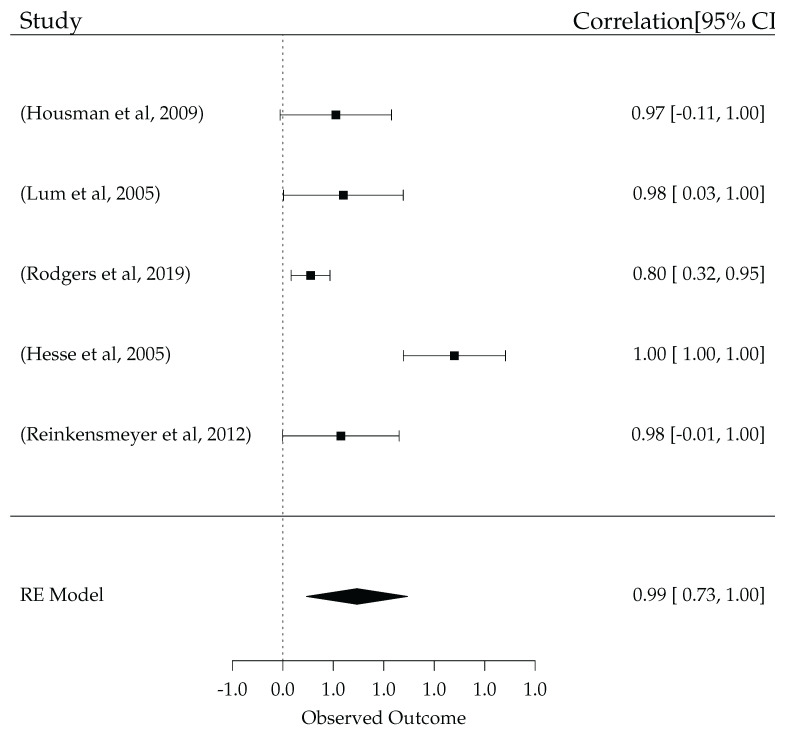
Pairwise comparison (Robot vs. Usual Care) [149,150,151,152,153].

MIME’s Randomized Controlled Trial was 15 Sessions long. Since the MIME has two modes of operation, they were tested as separate groups in addition to a group that used both modes of operation. The FM improvement in the unilateral therapy group was 7.9±1.91, the bilateral therapy group saw an improvement of 3.8±1.66, and the combined group’s FM improvement was 7.6±1.26. Comparatively, the control group’s improvement was 2.5±0.6 [151]. With these trials and their results, the forest plot in Figure 12 can give us an idea of the effect of robotic systems and how they compare to usual care. However, this is by no means a meta-analysis, as it does not account for any biases and is not extensive enough to be considered a meta-analysis, not to mention the randomized controlled trials are of various lengths and qualities but the forest plot is unweighted. It is merely to give us an idea of how these robotic systems compare up to their physiotherapy counterparts. For a more detailed analysis, see [155].

As seen in Figure 12, the effect of the robotic systems in the aforementioned RCTs, save for [152], was minimal. Moreover, that was the conclusion of [155], that “little evidence supported the superiority of experimented interventions over conventional rehabilitation”. However, they also state that some interventions were effective in *enhancing* poststroke motor recovery, rather than replacing traditional therapy.

### 5.3. Evaluation of the Current State of Robotic Rehabilitation Systems: The Gaps, Challenges, and Requirements

This section provides an attempt to answer the question of this paper: “Will your next therapist be a robot?”. To answer this question, a discussion on the current challenges and gaps in the robotic rehabilitation field is conducted. This section also suggests strategies to meet the requirements needed to achieve effective robotic rehabilitation systems. Based on the comprehensive analysis provided in this paper, it is clear that the current design of rehabilitation robots has focused more on the technical aspects of the robotic systems rather than the rehabilitation effectiveness of the variety of operational or training modes available to patients. For example, by comparing Table 3 and Table 4, it is clear that fewer systems have adopted multiple therapy modes. Instead, the focus was to increase the mechanical complexity of the robotic systems by increasing the number of sensors, degrees of freedom, and motors. However, the requirements of a successful clinical rehabilitative robotic experience are more than purely technical-based developments. The authors in [156] suggested that the aspects of the therapeutic psychology, usability, and ergonomics, in addition to the medical aspects, must also be addressed. The design of robotic systems for rehabilitation must be compatible with other elements developed by the therapist. Moreover, the design of a rehabilitation robot should meet the goals of the session instructed by a therapist. Task allocation is another critical aspect that should be considered when designing a robotic system. A good robotic therapy experience should be able to determine the best task allocation strategy by efficiently assigning tasks to be accomplished by the robot, the patient, or both of them.

Rehabilitation robotic solutions are recommended to be built by considering sensorimotor methods. These methods can help in reducing faulty motor behavior using proprioceptive and cutaneous stimulations [157]. To the best of the authors’ knowledge, the literature lacks robotic rehabilitation techniques that adopt proprioceptive and cutaneous stimulations. More research is encouraged in this field. The literature shows that several robotic solutions have utilized biomechanical methods to assist patients recover motor control. However, some important training modes including active and passive stretch and isometric exercise with/without resistance have not been widely implemented within robotic rehabilitation systems. Such exercises can benefit a number of patients with upper extremity disorders. A few works have considered physical agent modalities in the design of their robotic systems [158,159]. Adopting physical agent modalities can help in enhancing the performance of rehabilitation systems. For instance, heat can be utilized to reduce muscle spasms, cure joint stiffness, increase motion, and increase blood flow. On the other hand, electrical modalities can be utilized to train muscles, increase motion, and reduce pain.

Based on the discussion of this section and this paper, the short answer to the question: “Will your next therapist be a robot?” would be no, at least for the next decade for a fully dependent robotic system. Even though it is shown throughout this paper that robots have been effectively integrated into the rehabilitation field, it is clear that there are still some challenges to achieve an entirely independent robotic rehabilitation system. The following aspects need to be addressed and studied in future works:

- Cost: Many of the advanced robots used in rehabilitation are expensive, which limits their accessibility to patients;
- User-friendliness: Robots used in rehabilitation need to be easy to operate and require minimal training so that they can be used by patients with varying levels of physical and cognitive abilities;
- Adaptability: Robots need to be adaptable to various patient needs and abilities, which requires sophisticated algorithms and control systems;
- Safety: Robots must be safe to use, with built-in safety features to prevent accidents and injuries;
- Evidence-based: There is a need for more research to determine the effectiveness of robots in rehabilitation, and to identify the specific patient populations and conditions for which they are most useful;
- Ethical considerations: There are ethical considerations to be addressed, such as how to balance the benefits of using robots with the potential loss of human interaction and empathy.

## 6. Conclusions

This paper has presented a comprehensive review on robotic rehabilitation systems that have been recently proposed. Various robotic solutions have been presented in the literature with different configurations and approaches, from devices that simply make the patient repeatedly do an exercise to those that go as far as including enriched training environments to unlock the human brain’s true learning capabilities. Overall, when used in conjunction with traditional therapy, robots offer an enhanced experience for the patients. However, there is yet to exist a robotic system that completely replaces traditional therapy. The review had also discovered the advantages and disadvantages of various techniques used in robotic rehabilitation and pointed to the current gaps in the rehabilitation robotic field.

## Figures and Tables

**Table 2:** Comparison between therapy types.

	Active Therapy	Passive Therapy	Bilateral Therapy
Subtypes	Active Assistive	Passive Assistive	-
	Active Resistive	Continuous Passive Motion	
Advantages	- Efficient for advanced rehabilitation treatments	- Ideal for early stages of post-stroke symptoms	- Ideal for specific cases (e.g., Hemiplegia)
	- Has feedback information	- Easy to implement	- Simplicity
Disadvantages	- Needs patient interaction	- Does not get feedback from the patient	- Needs having some undamaged parts in the patient’s body
	- Can be complex to design	- Needs to be tuned continuously	- Only used for a few specific cases (e.g., hemiplegia)
Examples	[11,12,14,15,19,20,21,22,23,24,25,26,27,28,29,30,31,32,33,35,36,38,39,40,41,42,43,44,45,47,48,49,50,51,52,53,54,55,56,57,58,59,60,61,62,63,64,65,66,67,68,69,70,71,72,74,75,76,77,78,79,80,81,82,84,85,86]	[13,14,15,19,26,37,46,58,59,60,62,73,83]	[13,14,15,34,70]

**Table 3:** A summary of robotic rehabilitation systems based on the therapy type. CPM = Continuous Passive Motion; PA = Passive Assistance; AA = Active Assistance; BT = Bilateral.

Therapy	Movement	Paper	Year	DoF	Comment
CPM	Finger	[61,73],	2007, 2009	4,1	[59,73] are single-finger systems
	Shoulder	[95]	2022	2	Provides the movements of dorsiflexion (DF), plantarflexion (PF), abduction (AB), and adduction (AD).
PA	Sholder, Elbow, and Forearm	[46,83]	2006, 2005	5,5	[46,83] are Exoskeletons with Gravity Compensation technique
	Sholder and Elbow	[96]	2019	4	Upper-limb neurorehabilitation and treatment of spasticity
AA	Finger	[23,30,32,40,42,43,45,47,49,51,53,56,57,61,65,66,72,78,84,86,97,98]	2022, 2018, 2011, 2010, 2010, 2009, 2009, 2009, 2009, 2008, 2008, 2007, 2007, 2007, 2006, 2005, 2005, 2005, 2004, 2004, 2002, 1998	4, 1, 5, 2, 10, 6, 1, 4, 20, 6, 2, 8, 5, 3, 2, 1, 2, 4, 3, 7, 5, 5	Controlled Independently: [49,65,66,72,86,97]. Controlled Together: [30,32,43,56,98], Single Finger: [23,47,78]. The authors in [97] adopted four slider-crank mechanisms, each fixed, and with movement of one finger. In the reference [98]: A device consisting of a glove, a microcontroller, and a motor has been considered.
	Elbow	[29,33,35,41,52,69,70,79,82,85,99]	2018, 2009, 2008, 2007, 2007, 2005, 2004, 2003, 2001, 2000, 1999	[29] is 3, others: 1	[99]: The design consists of an array of pneumatically pressurized soft actuators, End-Effector systems: [29,33,35], others: Exoskeletons
	Wrist	[11,12,27,39,44,48,75]	2009, 2007, 2007, 2005, 2005, 1992, 1992	1, 1, 1, 1, 1, 5, 5	End-Effector systems: [11,12,27], others: Exoskeletons
	Shoulder	[76]	2003	2	Exoskeleton robot
	Shoulder and Elbow	[20,21,25,28,36,38,64]	2010, 2009, 2009, 2007, 2007, 2005, 2005, 2005	4, 2, 4, 3, 3, 5, 2, 2	All techniques have adopted Admittance Control
	Forearm and Wrist	[54]	2008	3	Exoskeleton robot
	Shoulder, Elbow, and Forearm	[24,67]	2012,2006,	6,3	All techniques have adopted Admittance Control
	Shoulder, Elbow, Forearm, and Wrist	[63,68,80,81]	2014, 2009, 2009, 2009	7,7,7,7	All techniques have adopted Admittance Control
AR	Forearm and Wrist	[50,74,100]	2018, 2008, 2007	1, 4,3	In the reference [100], an electromyography signal is used as an input to drive the joint movement [50] includes Elbow movement also
	Elbow, Forearm, and Wrist	[50]	2007	3	Exoskeleton robot
BT	Shoulder and Elbow	[34]	2007	2	End-Effector robot

**Table 4:** List of robotic rehabilitation systems capable of having multiple therapy strategies. CPM = Continuous Passive Motion; PA = Passive Assistance; AA = Active Assistance; BT = Bilateral.

Therapy	Movement	Paper	Year	DoF	Comment
CPM, AA	Finger	[59,62]	2009, 2009	6,2	[59] is a single-finger system
	Elbow	[58,60]	2009, 2009	1,1	[58,60] are Exoskeletons
	Shoulder	[19]	2015	7	Exoskeleton robot
	Forearm	[26]	2007	1	End-Effector robot
AA, AR	Elbow	[101]	2018	1	Surface EMG measurements are used to implement a force-based active and resistive control.
	Finger	[102]	2022	1	The device was actuated by six twisted string actuators (TSAs)
		[31]	2008	5	Have used Admittance and Impedance Control
	Elbow, Forearm, and Wrist	[55]	2008	4	Have used Admittance and Impedance Control
BT, CPM	Forearm, and Wrist	[13]	2003	1	End-Effector robot
BT, AA	Elbow	[70]	2001	1	Exoskeleton robot
AR, BT, AA, CPM	Shoulder and Elbow	[14,15]	2000, 2000	6,6	Have used Admittance and Impedance Control
PA, AA, AR	Arm	[103]	2020	6	Tested with an elderly female participant
	Wrist	[104]	2020	Up to 3	Robot consists of Series elastic actuators with high torque-to-weight ratios

**Table 5:** Advantages and disadvantages of known rehabilitation robots.

Robot	Advantages	Disadvantages
ETS-MARSE [19]	- Mimics natural human spine motion - Can be used for studying the biomechanics of the spine and testing spinal implants and surgical techniques	- Not designed for use in clinical rehabilitation settings - Expensive and complex to build and operate
Handcare [31]	- Designed specifically for hand and finger therapy - Lightweight and easy to use - Provides personalized and goal-oriented therapy	- Limited to hand and finger therapy only - Relatively new technology, may not be widely available
PNEU-WREX [46]	- Can assist with wrist and hand movements - Lightweight and easy to put on and take off - Has shown promise in improving upper limb function and independence	- Limited to upper limb therapy only - May not be suitable for individuals with severe upper limb impairments - Requires additional training for clinicians and therapists to use
surface Electro- MyoGraphy (sEMG) [76]	- Adaptive assistance that is natural and responsive to patient’s movements - Fine-tuned adjustments based on patient’s needs - Can learn and adapt to patient’s needs over time.	- Requires sophisticated control algorithms and sensors - May be expensive and complex to develop and maintain.
MyoPro (https://myomo.com/what-is-a-myopro-orthosis/, access on 25 April 2023)	- Provides powered assistance for upper limb movement - Easy to use	- Expensive - Limited evidence of efficacy
Rapael Smart Glove (https://www.neofect.com/us/smart-glove, access on 25 April 2023)	- Interactive training with haptic feedback - Wearable design	- Limited range of motion supported - Expensive
Harmony [17]	- Anatomically aligned shoulder mechanism - Unconstrained mobility of all joints - Supports body weight - Provides assistive force	- Expensive - Limited evidence of efficacy - Limited range of motion supported
ANYexo [18]	- Adaptable and customizable to different arm sizes and levels of assistance - Controlled by a smartphone app or joystick	- Experimental device not yet widely tested in clinical trials - Limited evidence of efficacy

**Table 6:** List of studies based on the type of the input signal.

Controller Input	Reference
Force/Torque	[13,14,15,19,21,36,39,79,80,81]
Optical Encoders	[14,15]
Position	[11,12,13,21,23,28,34,36,42,53]
Angular velocity	[11,12,33]
EMG	[27,35,41,45,52,58,60,62,63,65,66,68,70,72,75,76,78,82,85]
Joint angle	[26,27,29,42,43,47,48,51,59,64,69,72,86]
Cylinder pressure	[64]

**Table 7:** List of studies based on the actuator type used.

Actuator	Reference
DC	[11,12,14,15,20,23,25,27,30,31,34,35,36,37,38,41,42,45,47,48,51,52,57,59,61,62,63,66,68,70,72,76,77,79,80,81,82,84,85,86,87]
AC	[19,21,26,29,58,83,84]
Hydraulic	[60]
Pneumatic	[22,40,43,46,53,64,78]
FES	[36,56]

**Table 8:** Summary of the robotic rehabilitation systems based on the controller type.

Class	Technique	Paper	Year	Exp/Sim	Comment
PID	Optimized PID	[110]	2019	Sim	Controlling an exoskeleton of a three-DoF system designed to facilitate the movements of the elbow and the shoulder
		[111]	2015	Sim	Controlling a musculoskeletal system based on a five-DoF arm model and 22 muscles
	MIMO PID	[112]	2003	Exp	A trajectory control of a two-DoF wrist joint with neurologically intact subjects
	Linear PID	[113]	2010	Sim	EXO-UL7 robot
Robust	Fractional SMC	[114]	2020	Sim	Design of a 7 DoF upper limb robotic exoskeleton (u-Rob) which was controlled using fractional SMC
	Fuzzy SMC	[115]	2019	Sim	A seven-DoF upper-limb exoskeleton robot was controlled using Fuzzy SMC
		[116]	2015	Sim	A mechanical design of a new three-DOF exoskeleton robot for shoulder joint rehabilitation was also proposed. The parameters of the SMC controller were optimized using GA
Adaptive	ADRC	[117]	2014	Exp	The experiments were conducted on a model of a flexible joint robot, which imitates a real rehabilitation robot.
	NLADRC	[118]	2021	Sim	NLADRC and NLESO were adopted to track a sinusoidal path for a two-link model of an upper limb rehabilitation exoskeleton.
	ADRC	[119]	2022	Sim	LESO and FTSTD techniques were adopted to estimate the status of the system and to reject the disturbances.
	ADRC	[120]	2020	Exp, Clinical	ADRC and RESO were utilized to control a proposed rehabilitation device made from elastomeric materials.
AI-Based	EDRFNN model	[121]	2011	Sim	GA, HEP, and BP techniques were adopted to optimize the parameters of the model.
	RBF NN	[122]	2019	Exp	The proposed control system contained a disturbance observer with a radial basis function network

**Table 9:** The advantages and disadvantages of the control classes shown in Table 8 and discussed in Section 3.3.

Class	Advantages	Disadvantages	Papers
PID	- Simplicity	- Not optimal	[110,111,112,113]
	- Process independent	- Suffer from derivative noise amplification	
	- Acceptable performance with tuned parameters	- Needs tuning	
Robust control	- Advanced performance in the presence of bounded uncertainties and disturbances	- Cannot handle unbounded uncertainties and disturbances	[114,115,116]
	- Relatively Simple	- More complex than PID	
	- Stability can be proved using Lyapunov theory	- Chattering (for SMC controllers)	
Adaptive control	- Advanced performance in the presence of unbounded uncertainties and disturbances	- Not practical with large dimension systems	[117,118,119,120]
AI-Based control	- applicable to non-mathematical models	- Needs to be trained	[121,122]
	- Efficient in predicting models	- Good predictions need large data	
	- Non-linear nature	- Overfitting problems	

**Table 10:** Summary of robotic rehabilitation clinical trials. Notations: s = number of sessions, n = total number of participants, AS = at the end of the Study, AF = at follow-up.

		Housman et al. [150]	Lum et al. [151]	Rodgers et al. [149]	Hesse et al. [152]	Reinkensmeyer et al. [153]
Study Duration (Weeks)		8, s = 24	4, s = 15	12, s = 36	6, s = 30	8–9, s = 24
Follow-Up (Months)		6	6	6	3	3
n of Sex(m/f)	Control	7/7	4/2	101/153	12/10	12/1
	Experimental	11/3	2/3	101/156	12/10	5/8
Age	Control	56.4±12.8	59.9±5.5	62.5±12.5	64.0±11.6	61±13
	Experimental	54.2±11.9	72.2±11.7	59.9±13.5	65.4±11.5	60±10
Stroke	Control	8 ischemic, 5 hemorrhagic, 1 unknown	No Info.	214 cerebral infarction, 38 primary intracerebral haemorrhage, 2 subarachnoid haemorrhage	No Info.	4 ischemic, 6 hemorrhagic, 3 unknown
	Experimental	9 ischemic (1 with hemorrhagic conversion), 4 hemorrhagic, 1 unknown	No Info.	197 cerebral infarction, 58 primary intracerebral haemorrhage, 2 subarachnoid haemorrhage	No Info.	9 ischemic, 2 hemorrhagic, 3 unknown
FMA-UE (out of 66)						
Control	Baseline	18.1±5.0	26.0±4.72	45.47±11.48	7.3±3.3	22.9±7.4
	Change AS3	2.2±2.6	5.8±1.99	3.44±10.53	3.1±6.73	0.9±3.04
	Change AF4	1.5±2.7	13.8±2.77	5.94±10.13	9.3±14.53	0.1±3.04
Experimental	Baseline	21.7±5.9	39.2±6.08	45.47±10.89	7.9±3.4	24.1±8.8
	Change AS3	3.3±2.4	3.8±1.66	5.09±9.71	16.7±14.5	3.3±7.24
	Change AF4	3.6±3.9	7.4±1.98	6.14±10.39	22.1±16.5	2.4±6.93
Characteristics of experimental interventions used in clinical trials						
Robot		[83]	[14,15]	[11]	[13]	[46]
Robot Type		Exoskeleton	End-Effector	End-Effector	End-Effector	Exoskeleton
Degrees of Freedom		5	6	5	1	5
Control Strategem		Gravity Compensation	Admittance Control	Admittance Control	Admittance Control	Gravity Compensation
Type of Therapy		Passive	Bilateral	Active	Bilateral	Passive
Motor Learning Strategy		Assistance	Mirroring	Assistance	Mirroring	Assistance

## Data Availability

Not applicable.
